# High-resolution three-dimensional structural determination of unstained double-gyroid block copolymers through scanning transmission electron microscopy

**DOI:** 10.1038/s41598-023-39291-3

**Published:** 2023-07-26

**Authors:** Ying Chen, Jhih-Heng Yang, Ya-Ting Chang, I-Ming Lin, Chien-Nan Hsiao, Yeo-Wan Chiang, Chien-Chun Chen

**Affiliations:** 1 Department of Engineering and System Science, National Tsing Hua University, Hsinchu, 30013 Taiwan; 2 Department of Physics, National Taiwan University, Taipei, 106319 Taiwan; 3 Department of Materials and Optoelectronic Science, National Sun Yat-sen University, Kaohsiung, 80424 Taiwan; 4 Taiwan Instrument Research Institute, Hsinchu, 300092 Taiwan

**Keywords:** Materials science, Mathematics and computing

## Abstract

Block copolymer-based multicomponent materials have garnered considerable attention because of tunable properties due to their various constituents. The use of electron tomography through transmission electron microscopy (TEM) for the three-dimensional (3D) imaging of stained block copolymers is an established approach for investigating structure-property relationships. Recently, scanning transmission electron microscopy (STEM) with an annular dark-field (ADF) detector has emerged as a method for the 3D structural analysis of unstained block copolymers. However, because of a lack of electron contrast, only a few low-resolution 3D reconstructions were reported for light elements. Herein, we report the first 3D structural analysis of a 200-nm-thick film composed of unstained double-gyroid block copolymers-polystyrene-b-poly(2-vinylpyridine) (PS-P2VP)-at a resolution of 8.6 nm through spherical aberration Cs-corrected STEM. At this resolution, P2VP molecules can be distinguished from PS molecules in z-contrast 3D reconstructions obtained both experimentally and theoretically. The 3D reconstructions revealed structural differences between stained and unstained specimens.

## Introduction

Photonic crystals are materials with ordered structures that utilize tunable bandgaps to allow specific electromagnetic waves to penetrate while reflecting others. Various photonic crystal structures can be found in nature, such as in the feathers of peacocks, wings of butterflies, and skins of chameleons, and are used for courtship and reproduction, deterrence, and adaptation to changing environments, respectively^1–6^. Different from the approach of the chemical synthesis of brush copolymers^7^, a novel technique for the fabrication of double-gyroid block copolymer thin films involving the trapping of structural coloration (TOSC) generates tunable and reversible photonic bandgaps covering the range of visible light^8^.

Staining is a conventional protocol for the characterization of the 3D structures of block copolymers at the nanometer level; it helps enhance the phase contrast in TEM images of block copolymer structures. However, a stain may accumulate on the surface of specimens rather than being uniformly distributed throughout block copolymer films; moreover, evidence has suggested that staining induces slight changes in the film’s lattice geometry and degree of disorder^9–13^. Scanning-mode TEM, also called the z-contrast imaging, appears to be a promising approach for observing the native 3D structures of polymer films^14^.

Compared with the phase alteration of a TEM image, the contrast of a STEM image is much simpler to interpolate. Studies regarding the STEM tomography of polymers have focused on low-dose schemes to reduce radiation damage^15–17^. With the advancements in reconstruction algorithms, only a few dozen projections are required for 3D reconstruction^14,18–20^. However, because of the inadequate scattering efficiency of light elements, only low-resolution images generate a sufficient signal-to-noise ratio for reliable 3D reconstruction.

Studies have reported that Cs-corrected STEM tomography with an iterative Fourier-based algorithm is useful in achieving the atomic resolution required for 3D reconstruction^21,22^. However, radiation damage, carbon contamination, and sample drifting may reduce the quality of acquired images^23^. Moreover, the samples must be either particle-like or needle-shaped materials, rather than thin films, to alleviate the effects of background noise and the inconsistencies among projections at different orientations.

To overcome the aforementioned shortcomings, we developed a novel approach that combines small-spot-size Cs-STEM, an unsupervised denoising technique^24^, and a Fourier-based iterative algorithm to visualize the contrast difference between PS and P2VP molecules in 3D reconstructions at a resolution of 8.6 nm.

## Sample preparation and data analysis

Bulk samples were prepared through solution casting using 1,1,2-trichloroethane (TCE) at room temperature. The samples were dissolved in TCE at 5 wt%. The solution was placed in a 6-mL vial, which was sealed tightly with aluminum foil. After the samples had dissolved completely, the aluminum foil was removed to allow the solvent to evaporate. To minimize the formation of defects and grain boundaries, the solvent was evaporated slowly at room temperature. The bulk samples were embedded in epoxy and sectioned using an ultramicrotome (Reichert EM FC7) at room temperature to prepare samples for TEM observations. The sectioned samples were stained through exposure to $RuO_4$ vapor for 60 mins. After staining, the P2VP domains appeared dark in the conventional TEM images, whereas the PS domains appeared light.

A STEM experiment was performed using an FEI Titan microscope under the following conditions: energy of 200 keV; spherical aberration of 58 nm; spot size of 9; illumination semiangle of 25 mrad; camera length of 135 mm; inner high-angle ADF (HAADF) detector angle of 35.9 mrad; and outer HAADF detector angle of 143.6 mrad. Gold particles (30 nm) were deposited on the Polystyrene-b-poly(2-vinylpyridine) (PS-P2VP thin film^25^. Projected images were acquired by rotating an advanced tomography holder (Model 2020; Fischione Instruments) from − 60° to 60° with an angular increment of 2°. Selected areas were monitored during rotation through the tracking of a single gold nanoparticle.

**Figure 1:**
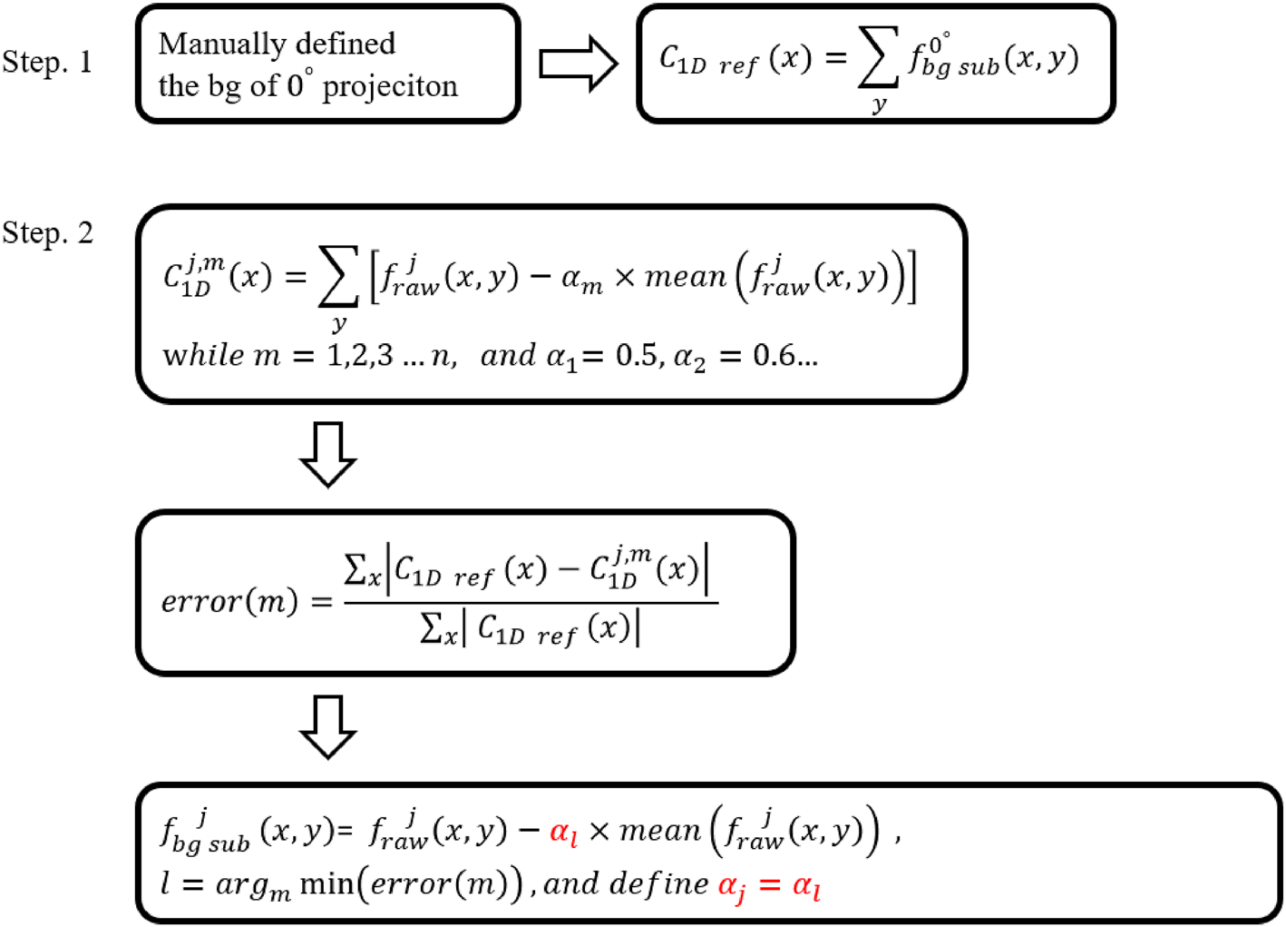
The schematic of background subtraction. While the $C_{1 D \text{ ref } }$ is the $1 \textrm{D}$ curve of 0-degree projection after manually selecting the background value, as a reference to the other degree. $C_{1 D}^{j, m}$ is the $1 \textrm{D}$ curve of the $\textrm{j}{\textrm{th}}$ projection after subtracting the $m^{\text {th}}$ $\alpha$, and $\alpha _l$ is defined by the minimum error (*m*).

To evaluate the robustness of STEM tomography, a stained 200-nm-thick PS-P2VP thin film was used for comparing 3D STEM and TEM images. Tilt series of projections of the same area were acquired through STEM and TEM. Because the field of view was fixed during rotation, the projections were cropped in the direction perpendicular to the rotation axis (i.e., the width of the image) on the basis of the cosine of the rotation angle. Next, the schematic of background subtraction is represented in Fig. 1. The first step in this process is to select an initial cutoff value for the 0-degree projection by monitoring the density distribution within the sample area. If negative values are detected within the sample area, the cutoff value is iteratively decreased until no negative densities remain. Then, we summed along the $\textrm{y}$-axis to obtain a $1 \textrm{D}$ curve of 0 -degree projection ($C_{1 D \text{ ref }}=\sum _y f_{\text{ raw }}^j(x, y)$, where the $\textrm{x}$-axis is the rotation axis), after the background subtraction as the reference for other background subtraction. In the second step, the background subtraction factor $\alpha _j$ [As shown in Eq. (1)] will be decided. When the $1 \textrm{D}$ curve of the $\textrm{j} {\text{ th }}$ projection after subtracting the $\textrm{m} {\text{ th }} \alpha$ is calculated (i.e., $C_{1 D}^{j, m}$ ), we monitor the error by surveying all available $\alpha _m$, and $\alpha _l$ is determined when the error is minimized. Finally, defining $\alpha _l$ as $\alpha _j$ for the factor of $\textrm{j}{\text{ th }}$ projection.

1 $$\begin{aligned} f_{bg sub}^j(x, y)=f_{\text {raw }}^j(x, y)-\alpha _j \times {\text {mean}}\left( f_{\text {raw }}^j(x, y)\right) \end{aligned}$$

The $f_{\text{ raw } }^j$ is the raw $j{\text{ th } }$ projection, whereas the $f_{bg sub}^j$ is the *j*th projection after background subtraction.

Fourier interpolation^26^ was then performed to construct a 3D Fourier space with Cartesian coordinates from the 2D projections, as shown in Eq. (2).

2 $$\begin{aligned} F\left( k_x, k_y, k_z\right) ={\sum _{i}} {f_{o b s}^{j}}\left( x_i, y_i, z_i\right) e^{-2 \pi i\left( k_x x_i+k_y y_i+k_z z_i\right) } \quad \forall D_j<D_{t h} \end{aligned}$$

where $D_j$ is the distance from the interpolated voxel to the *j* *th* projection; $D_{th}$ is defined as the threshold distance. $f^{j}_{obj}(x_{i},y_{i},z_{i})$ presents the 2D projections in 3D Cartesian coordinates.

After all available 3D Fourier grids are calculated by Eq. (2), the iteration is then conducted back and forth between the Fourier and real spaces. In Fourier space, the available grids are fixed during the iteration. In real space, a support is determined to constrain the sample area, and the grids with negative density are removed as well. The iteration is terminated when no improvement can be observed.

**Figure 2:**
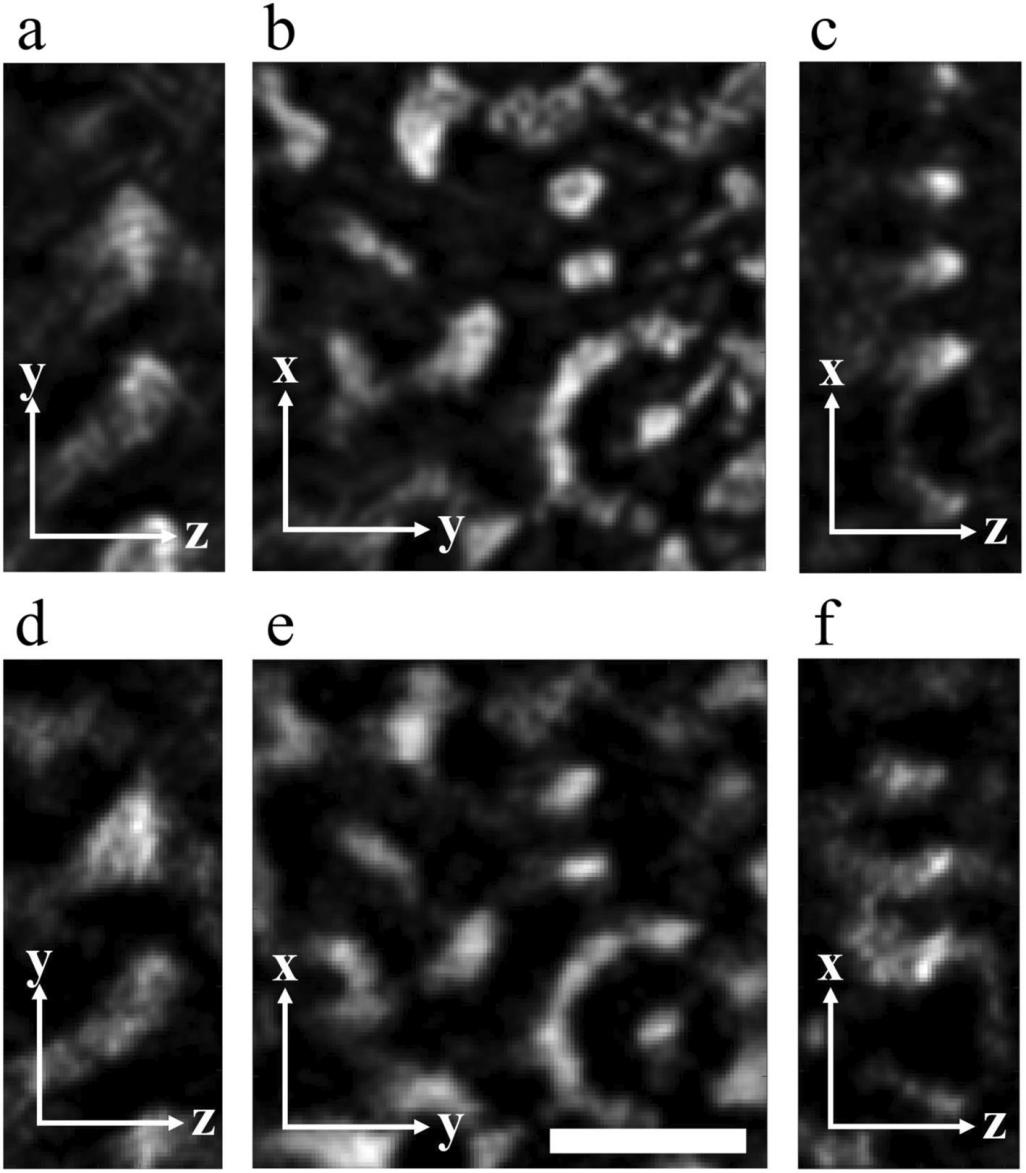
Comparison of 3D TEM and STEM reconstructions of stained PS-P2VP. (**a**–**c**) Selected YZ, XY, and XZ slices of the 3D TEM reconstruction. (**d**–**f**) Same YZ, XY, and XZ slices of 3D STEM reconstruction. The scale bar is 200 nm.

## Results and discussion

Figure 2 presents the images of the reconstructed YZ, XY, and XZ slices (with the beam direction along the z-axis; thickness, 4.3 nm) obtained through TEM and STEM tomography. With staining, the reconstructions revealed consistent inner density distributions, indicating that STEM tomography can achieve reliable reconstruction similar to that achievable through conventional TEM tomography. Notably, the density distribution along the z-axis was more uniform in the STEM reconstruction than in the TEM reconstruction. Stain accumulated on the surface resulted in severe phase contrast in TEM mode, whereas z-contrast imaging (i.e., STEM mode) avoided this artifact.

After the robustness of STEM tomography was validated, this approach was adopted to explore unstained block copolymers. Under the same experimental conditions, a total of 61 projections from − 60° to 60° (40,000 × magnification) were collected from an unstained 200-nm-thick PS-P2VP thin film. An unsupervised denoising technique, empirical mode decomposition (EMD), was employed to increase the signal-to-noise ratio^24,27–29^. After decomposition, we removed the first intrinsic mode function, which corresponded to the fluctuations with the shortest length scale and was thus unlikely to contain true structural information.

**Figure 3:**
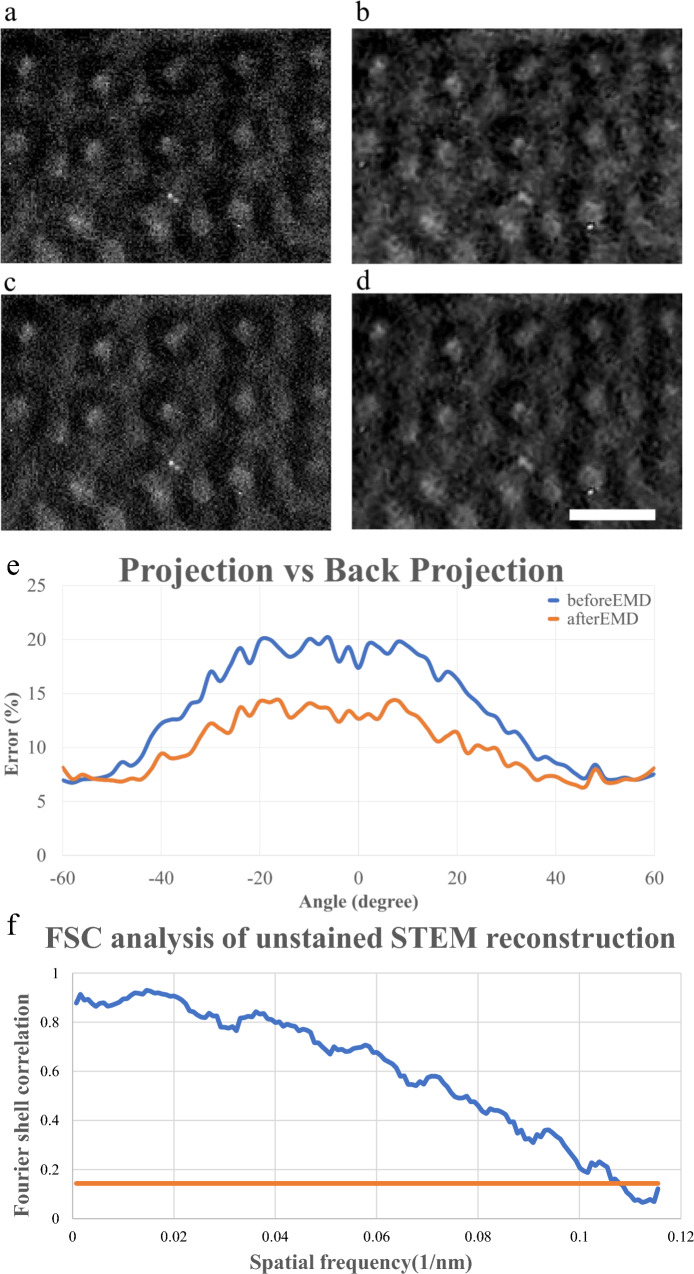
Raw and reconstructed projections with and without denoising. The figure shows the 0° projections (**a**) before and (**b**) after EMD as well as 0° backprojections of the 3D reconstruction (**c**) before and (**d**) after EMD. The scale bar is 200 nm. (**e**) Error distribution between projection and backprojection with EMD (orange) and without EMD (blue) preprocessing. (**f**) The FSC analysis of unstained STEM reconstruction (blue) and 0.143 cutoff (orange).

Figure 3a and b depicts the raw and denoised projections, respectively. The interconnections of the block copolymer skeletons reveal lower levels of fluctuation after EMD denoising. To qualitatively compare the reconstructions, we performed backprojections from the reconstruction (Fig. 3c,d) and calculated the error between the projections and backprojections (Fig. 3e). The average error (*Err*(*j*)) without EMD was 13.4%, whereas that with EMD was 10.1%. The Fourier shell correlation (FSC) analysis (Fig. 3f) is calculated by comparing the reciprocal spaces generated from the given projection and the reconstruction.

3 $$\begin{aligned} {\text {Err}}(j)=\frac{\left| \sum _x \sum _y\left( f_{\text{ given } \text{ proj } }^j(x, y)-f_{\text{ back } \text{ proj } }^j(x, y)\right) \right| }{\sum _x \sum _y f_{\text{ given } \text{ proj } }^j(x, y)} \end{aligned}$$

The $f_{\text{ given } \text{ proj } }^j$ represents the $j{\text{ th } }$ given projections and $f_{\text{ back } \text{ proj } }^j$ is the $j{\text{ th } }$ back projections calculated from reconstruction.

The chemical formula for $\textrm{PS}$ (Polystyrene) is $\textrm{C}_8 \textrm{H}_8$ with a molar mass of $104.152 \,  \mathrm {g} / \textrm{mol}$, while the chemical formula for $\textrm{P} 2 \textrm{VP}$ (Poly(2-vinylpyridine)) is $\textrm{C}_7 \textrm{H}_7 \, \mathrm {N}$ with a molar mass of $105.14 \,  \mathrm {g} / \textrm{mol}$. By multiplying the density of each substance (PS $\left( 1.05\,  \mathrm {g} / \textrm{cm}^3\right)$ and P2VP $\left( 1.15 \,  \mathrm {g} / \textrm{cm}^3\right)$), we obtain a ratio of 1.106. A distinct difference can be told when PS and P2VP are accumulated. Therefore, in the projections in Fig. 3, the density difference is observable. To validate the reliability of this result, we clustered the reconstructed densities into two groups, benzene rings with and without the aforementioned nitrogen atom, and performed multislice simulation under the assumption of the uniform distribution of all molecules within the 4.3 × 4.3 × 4.3 nm^3^ voxels. The multislice simulation was performed using Dr. Probe under the following parameter settings: energy of 200 keV; spherical aberration of 58 nm; illumination semiangle of 30 mrad; inner angle of the HAADF detector of 35.9 mrad; and outer angle of the HAADF detector of 143.6 mrad. When a voxel of 4.3 nm is considered, the voxel of P2VP generates approximately 10.8% higher contrast than does that of PS.

**Figure 4:**
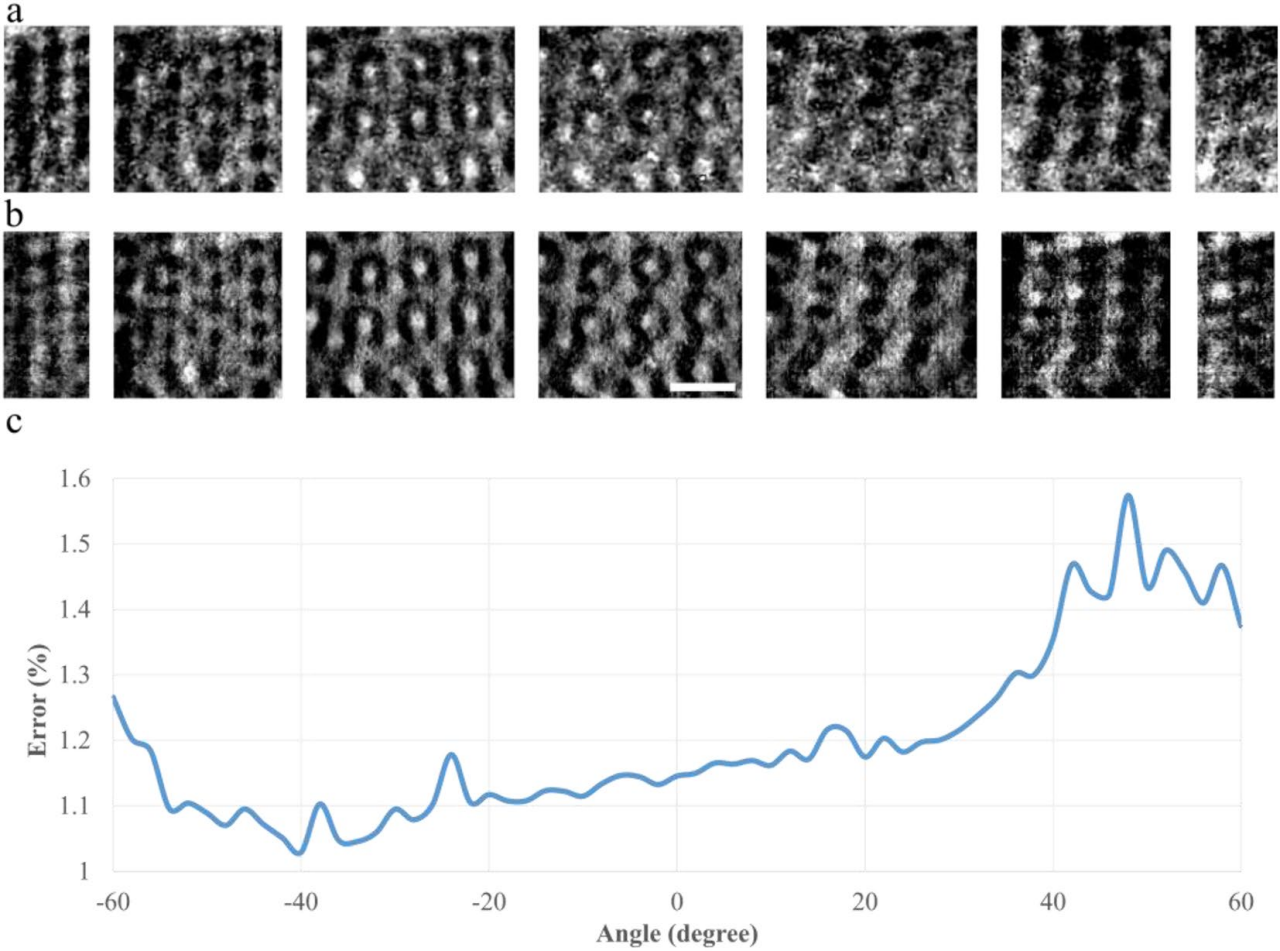
Experimental and simulated projections. (**a**) Raw projections at − 60°, − 40°, − 20°, 0°, 20°, 40°, and 60°. (**b**) Simulated projections at − 60°, − 40°, − 20°, 0°, 20°, 40°, and 60°. (**c**) The error between the two projections at all angles. Scale bar is 200 nm.

We replaced the aforementioned two-cluster voxels with the values of 1.108 and 1 to construct a 3D binary model. The 3D model was backprojected to generate 2D projections from all 61 angles. Figure 4 presents the experimental and theoretical projections and their calculated differences. The average error of approximately 1.2% suggested high consistency between the experimental and theoretical projections.

4 $$\begin{aligned} \Theta =2.75\left( \begin{array}{r} \sin (2 x) \times \sin (z) \times \cos (y) \\ +\sin (2 y) \times \sin (x) \times \cos (z) \\ +\sin (2 z) \times \sin (y) \times \cos (x) \end{array}\right) -1\left( \begin{array}{r} \cos (2 x) \times \cos (2 y) \\ +\cos (2 y) \times \cos (2 z) \\ +\cos (2 z) \times \cos (2 x) \end{array}\right) -0.2 \end{aligned}$$

**Figure 5:**
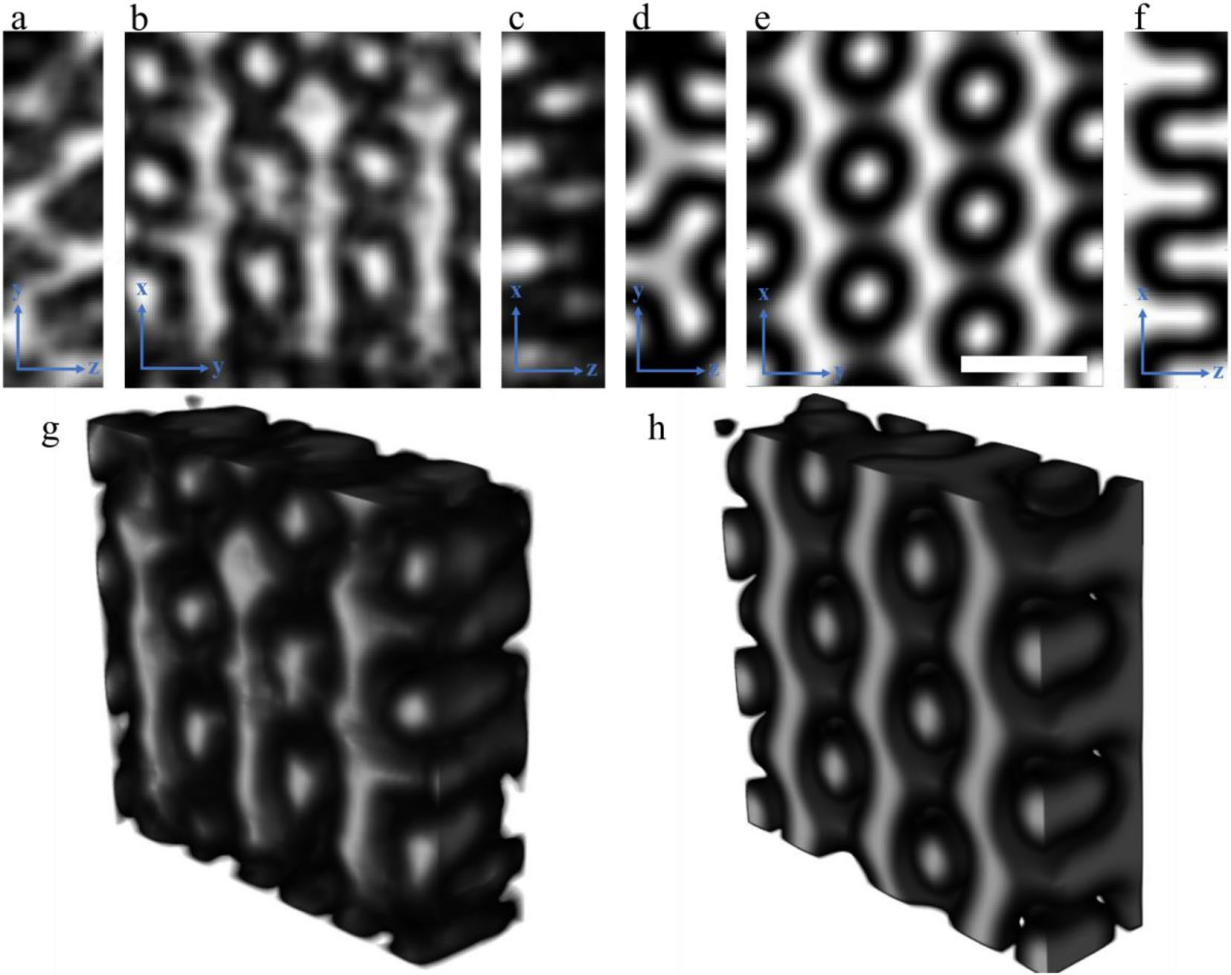
3D reconstruction and mathematical model of double-gyroid PS-P2VP. (**a**–**c**) Selected YZ, XY, and XZ slices of the reconstruction. (**d**–**f**) Same YZ, XY, and XZ slices of the mathematical model. (**g**, **h**) Isosurface of the 3D reconstruction and model indicating the consistency of cross-sections in three perpendicular directions. The scale bar is 200 nm.

A standard PS-P2VP model was constructed using the formula [i.e., Eq. (3)] of the double gyroid. Figure 5 depicts the 4.3-nm-thick central YZ, XY, and XZ slices corresponding to the $(110),(1 \overline{1} 1)$, and $(\overline{1} 12)$ planes, respectively. The periodic structures in (110), the Y-shaped graphics in $(1 \overline{1} 1)$, and the crossed-comb patterns in $(\overline{1} 12)$ can be recognized in all slices and in the cross-section of the 3D reconstruction.

**Figure 6:**
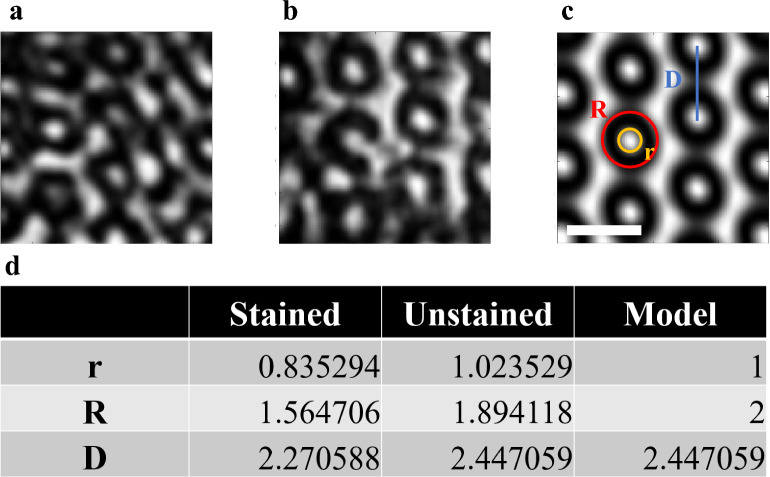
Measured structural parameters of stained and unstained reconstructions and mathematical models. The XY slice of (**a**) stained PS-P2VP, (**b**) unstained PS-P2VP, and (**c**) the mathematical model are presented. (**d**) The average inner(*r*), and outer diameters(*R*) and the interval between circles(*D*) of the reconstructions and model were calculated from selected local areas. The unstained reconstruction is more consistent with the model. The scale bar is 200 nm.

The PS–P2VP block copolymer structure is composed of two types of domains staggered in a film rather than stacking PS and P2VP films. Each segregation leads to the nanoscale to sub-microscale ordered patterns, namely, microphase separation, the size of a PS and a P2VP film can not be well-defined. Figure 6, A better structural comparison of PS and P2VP domains needs to be performed along a specific zone direction. Since the size varies, it will be more applicable if we normalize the parameters based on a specific length. Here we assume the inner radius of the model is 1 and calculate other parameters accordingly.

## Conclusion

In conclusion, our results demonstrate the robustness of high-resolution electron tomography for imaging stained or unstained block copolymers. STEM provides sufficient z-contrast for low-z specimens, without any staining requirement. Furthermore, EMD preprocessing improves the signal-to-noise ratio of projections. The adoption of an iterative Fourier algorithm may help to optimize tomographic reconstruction with missing wedges. The projection reconstructed in this study was similar to the accepted structure of double gyroids. Thus, this technique may be useful for the evaluation of other low-z specimens or block copolymer systems.

## Data Availability

The datasets used and/or analyzed during the current study available from the corresponding author on reasonable request.

## Code availability

All of our image reconstruction and data analysis source codes will be freely available immediately after this work is published.
